# Understanding barriers and facilitators to non-pharmaceutical chronic pain research engagement among people living with chronic pain in the UK: a two-phase mixed-methods approach

**DOI:** 10.1136/bmjopen-2024-089676

**Published:** 2024-12-22

**Authors:** Kirralise Hansford, Anna E Crossland, Daniel Baker, Catherine Preston, Kirsten J McKenzie

**Affiliations:** 1 University of York, York, UK; 2 Department of Psychology, University of York, York, UK; 3 University of Lincoln, Lincoln, UK

**Keywords:** QUALITATIVE RESEARCH, Patient Participation, Chronic Pain

## Abstract

**Abstract:**

**Objectives:**

Chronic pain treatment engagement is dominated by pharmaceutical methods, while previous research has assessed barriers to uptake of non-pharmaceutical treatments, there has not been research one step earlier in the treatment development pipeline; assessing barriers to take part in research that develops non-pharmaceutical chronic pain treatment methods.

**Design:**

A two-phase approach was used to assess barriers and facilitators to research participation for people living with chronic pain. Online focus groups were run in phase 1, generating qualitative data, while phase 2 used the themes identified within phase 1 to assess agreement and disagreement.

**Setting:**

Participants consisted of people with chronic pain across the UK.

**Participants:**

36 participants with chronic pain conditions (defined as any pain lasting or recurring for more than 3 months) were recruited for phase 1. Seven participants could not attend their focus group or a subsequent session, leaving a final sample size of 29 participants (83% female, 17% male; age=20–78 years, M=44.3 years). Phase 2 consisted of 103 participants (89% female, 10% male, 1% prefer not to say; age=20–80 years, M=46.6 years).

**Results:**

Phase 1 identified the largest barrier to be ‘distrust’, relating to a distrust of medical and research professionals, distrust of confidentiality assurances and distrust that the research would have an impact. The greatest facilitator identified was ‘improved accessibility’, which related to the accessibility of the research environment, the type of research being conducted and accessible advertisement of the research within trusted settings. Phase 2 found around 80% agreement with all facilitator themes and a mix of opinions regarding barrier themes, highlighting the individuality of barriers experienced when living with chronic pain.

**Conclusions:**

Addressing the barriers and implementing the facilitators identified here ensures that patient participants are comfortable and safe within research environments. Furthermore, this project provides recommendations for researchers to follow to help increase patient engagement in research studies.

**STRENGTHS AND LIMITATIONS OF THIS STUDY**

- The mixed-methods approach allowed for in-depth qualitative analysis supported by quantitative measures.
- Different data collection methods (online focus groups and short online questionnaire) allowed for flexibility of participation options.
- A limitation of the sample was the relatively homogeneous sample demographics; predominantly white and female.
- A key methodological limitation was the use of online measures, which excluded individuals without access to the internet.

## Introduction

 Chronic pain, defined as pain lasting for more than 3 months,^1 2^ is increasingly treated with non-pharmaceutical methods (without drugs/surgery) due to evidence that pharmaceutical treatments alone can be unsafe and/or ineffective.^3 4^ However, the use of pharmaceutical treatment is still prevalent with 25% of people in the USA relying solely on pharmaceutical methods for chronic pain management,^5^ 96% of people with chronic pain in the UK receiving an opioid analgesic between 2004 and 2009,^6^ and despite National Institute for Health and Care Excellence guidelines (GID-NG10068) advising against pharmaceutical chronic pain treatments, prescription rates for pain killers are reportedly unchanged for years.^7^ Research concerning the lack of uptake of non-pharmaceutical treatments has found that barriers include high costs, transportation problems, low patient motivation, healthcare appointment time conflicts and concerns about non-pharmaceutical treatment efficacy.^8^,^11^ However, no investigation has yet explored barriers and facilitators for participants taking part in research into non-pharmaceutical treatments, which is vital for their successful development and uptake.

In 2018, national standards were set for patient engagement in research highlighting the importance of including patients when designing novel treatments.^12^ Patient engagement can lead to improved credibility of results and improvements in direct applications to patient samples.^13^ Therefore, understanding barriers and facilitators for patient involvement is paramount. However, achieving meaningful patient involvement in research and clinical trials can be a difficult process, with reports of around 37% of projects having a reduced sample size than planned and 11% of clinical research failing to recruit any participants at all.^14^ Additionally, the people who do participate in research are often from homogeneous demographics, with a 2021 report of people who take part in health research in the UK finding that participants are predominantly female, white, heterosexual and aged over 61 years.^15^ Previous work has found barriers to patient involvement in chronic pain research and/or clinical trials to include practitioners’ lack of knowledge about conditions, poor communication, lack of knowledge about the nature of clinical trials, concerns about adverse side effects, misgivings relating to being used as ‘guinea pigs’ and distrust of the medical community.^16 17^ Research assessing facilitators for people with chronic pain taking part in research has found motivators to be social engagement/enjoyment, pain improvement/advancement of science, to seek relief of pain (both short term and long term), to try a different drug, to have their pain taken seriously, and to receive compensation for taking part.^18 19^

This study, therefore, aimed to address the apparent gap in literature by assessing the barriers and facilitators to participation specifically in non-pharmaceutical research. Phase 1 used a nominal group technique (NGT), commonly used to assess barriers and facilitators within online focus groups. Following this, phase 2 extended on previous methods by using an online questionnaire to assess agreement/disagreement with the barriers and facilitators identified within phase 1 in a much larger sample, to assess the generalisability of the facilitator and barrier themes to the wider UK population with chronic pain. For phase 1, our preregistered hypotheses were based on the most prevalent barriers mentioned in existing literature, which were that (1) transportation problems and (2) the concerns regarding the efficacy of non-pharmaceutical treatments, would arise as top-most barriers. Since previous research was more limited for facilitators to take part in research, there were no directional hypotheses regarding facilitators. We had no directional hypotheses for phase 2.

## Manuscript structure

This study comprises two phases, methods and results for phase 1 will be presented first ("Phase 1 Methods"; "Phase 1 Results"), then methods and results for phase 2 will be detailed ("Phase 2 Methods"; "Phase 2 Results"), with discussion of both phases included in the general discussion ("Discussion").

## Triangulation

Triangulation between the qualitative and quantitative data within this report was approached sequentially at the design stage, whereby the findings from the qualitative data in phase 1 were used to create materials for the quantitative data in phase 2. Qualitative and quantitative data were then merged during the manuscript’s discussion and integrated via written narrative to allow for a holistic approach to understanding which barriers and facilitators exist for people when considering taking part in non-pharmaceutical chronic pain research.

## Phase 1 methods

### Preregistration

The preregistration for this study can be found on OSF at the following DOI: https://doi.org/10.17605/OSF.IO/37SNZ. Slight deviations from the preregistration consist of the following:

- Phase 1 sample size aim was 30 participants minimum, and although 36 participants were recruited for phase 1, only 29 were able to take part in a focus group session.

### Participants

36 participants with chronic pain conditions (defined as any pain lasting or recurring for more than 3 months) were recruited from the UK through contact with UK-based chronic pain support groups and online advertisements to take part in an online focus group. The sample size aim of 30 participants was derived from previous studies employing the same NGT or similar structured interviews, which recruited between 18^11^ and 68^9^ pain patients, with most studies recruiting around 25.^8 10 20^ Therefore, this sample size aligns with the upper end of the average sample size. Seven participants could not attend their focus group or a subsequent session, leaving a final sample size of 29 participants (83% female, 17% male; age=20–78 years, M=44.3 years). Demographic information for phase 1 participants can be seen in table 1. A list of the chronic pain conditions within this sample can be seen in online supplemental table S1. It was determined in advance that data collection would finish once the desired sample size was met, and not when data saturation was achieved.^21^

**Table 1:** Ethnicity and highest education level achieved for phase 1 participants

Ethnicity		Education level	
White	79%	Undergraduate degree	31%
Asian or Asian British	7%	College (A-level equivalent)	31%
Mixed/multiple ethnic groups	7%	Master’s degree	17%
Black, Black British, Caribbean or African	3%	Postgraduate PhD or MD	17%
Other ethnic group	3%	Primary school	3%
		Secondary school	0%

### Patient and public involvement

Patients and the public were not involved in the design, or conduct, or reporting, or dissemination plans of our research. Public chronic pain groups were contacted to help with the advertisement of the research.

### Data collection

An NGT was used to assess barriers and facilitators to participation in non-pharmaceutical chronic pain research. An overview of the NGT has been reported by Harvey and Holmes,^22^ who recommend using face-to-face focus groups to obtain the views of experts on a given topic and bring about group consensus. Here, the experts were people with lived experience of chronic pain. It is recommended that focus groups for the NGT are to be run with between 5 and 9 participants,^20^ however, due to participant dropout, groups were run with between 2 and 7 participants. All focus groups were conducted online using Zoom video conferencing software (Zoom Video Communications, San Jose, California, USA), V.5.17.7.

Focus groups were conducted by authors KH (lead researcher), CP (supervisor) and AEC (researcher), all researchers conducting focus groups were female. No relationship between the researchers and the participants was established prior to commencement of the focus groups. No additional people were present during the focus groups other than the researchers and the participants. Participants were provided with a brief background to the researchers themselves and to the study, and an explanation of key terms and procedures. The researchers did not declare any bias regarding their reasons for interest in the research topic. Distinctions were made between pharmaceutical and non-pharmaceutical chronic pain treatments before the first question was presented to the group. Participants were asked about barriers to their participation in non-pharmaceutical chronic pain research; ‘What are some barriers to patients participating in research about chronic pain? In other words, why do some patients not want to participate or what makes it hard for them to participate?’ along with a question regarding facilitators of participation in non-pharmaceutical chronic pain research; ‘What are some of the things that would make it more likely for patients to participate in chronic pain research? What makes it easier for patients to participate?’ These questions were adapted from previous research using NGT to investigate barriers and facilitators to using non-pharmacological pain treatments and were presented in a random order for each group to remove ordering bias.^8^ After each question, participants were asked to silently write down as many responses to the question as possible in 5 min before the researcher asked each participant to say their responses aloud, or type using the chat function. The researcher wrote each response on an editable document (Google Forms, Google, Mountain View, California, USA) until each participant had all their answers recorded. Group discussion was encouraged to clarify any responses, and edit as necessary, in addition to consolidating any answers that the participants deemed to be identical or very similar. Focus groups lasted around 60 min.

Once the final list of answers was agreed on, each participant was sent a link to the Google Form and anonymously voted on the most important (3 points), second most important (2 points) and third most important item (1 point). Researchers were blind to which response was given by which participant. The same process was completed for both the barriers and the facilitators question, after which the researchers tallied up the voting, giving the topmost barriers and facilitators for each focus group.

Phase 1 data from all focus groups can be seen on the following OSF page: https://osf.io/8y7rz/.

### Data analysis

To facilitate comparisons of items from each focus group across all sessions, researchers used thematic analysis through a deductive approach^23^ to categorise items into either facilitator or barrier themes based on a consensus of interpretation of the item meanings. A thematic analysis approach can be employed when generating large data with the NGT,^24^ therefore, this was the approach used here. A post-positivist approach was used for thematic analysis, to focus on the individual experiences of people living with chronic pain when considering taking part in non-pharmaceutical research.^25^ Through these approaches, three researchers (KH, AEC and CP) independently reviewed the raw data for familiarisation, before identifying patterns within the data set. They then created codes for each group of items, before placing these coded items into overarching themes within either the barrier or facilitator structure. An additional fourth researcher (KJM) was included to facilitate the discussion of theme review among the three researchers. Following this, two researchers (KH and CP) refined and defined the themes with the following agreements; barrier themes percentage agreement: 87.03%,^26 27^ barrier themes Cohen’s kappa: 0.832 (strong agreement^28^; near perfect agreement^29^), facilitator themes percentage agreement: 89%,^26 27^ facilitator themes Cohen’s kappa: 0.869 (strong agreement^28^; near perfect agreement^29^). This process resulted in the final list of themes agreed on by all four researchers. Themes were sorted by size, with the theme containing the most items presented first. Participant ratings of items were tallied to give the topmost items within each theme.^24^

## Phase 1 results

From the 7 focus groups, 121 items were generated for barriers and 95 items were generated for facilitators. As a result of thematic analysis, seven barrier themes were created and can be seen in online supplemental table S2: (1) distrust; (2) lack of accessibility/physical practicalities; (3) chronic symptoms and comorbidities, (4) lack of information, (5) lack of motivation, (6) self-identification/eligibility and (7) cultural barriers/individual differences. Online supplemental table S3 shows the five facilitator themes that were created: (1) improved accessibility, (2) positive impact of participation, (3) detailed and accessible information, (4) motivation and (5) safe space.

Online supplemental tables S2,S3 show the themes identified, with the themes (and wherever relevant, subthemes) listed according to size, with the theme containing the most items listed first. All items from the focus groups are included within their associated theme, and the highest rated items by participants during the ranking section of the NGT are denoted with superscript text. Since items are collated across all focus groups, several themes contain more than one highest-ranked item.

The largest barrier theme from phase 1 was ‘distrust’, consisting of subthemes, ‘anonymity/confidentiality’, ‘impact of research’ and ‘professionals/setting’. This theme can be described as participants having a distrust of the level of anonymity and confidentiality within research or having a distrust of medical or research professionals. Within the subtheme ‘anonymity/confidentiality’, participants reported a ‘lack of trust of confidentiality of data’ and expressed not wanting to share medical/personal information with research staff. Within the subtheme ‘impact of research’, top-ranked barriers were ‘not knowing if the research will benefit me or others’ and ‘not being sensitised to the importance of such research’. The subtheme ‘professionals/setting’ consisted of the top-ranked barrier ‘lack of understanding about chronic pain’ with other high-ranking barriers related to a fear of scrutiny; group situations; and of what might be involved in research, along with expressions of previous negative experiences.

The next largest barrier theme was ‘lack of accessibility/physical practicalities’, which consisted of subthemes ‘travel’, ‘accessibility—personal’, ‘accessibility—technological’ and ‘time’ and can be characterised as a lack of logistical practicality when taking part in research, such as inaccessible physical locations, limited travel options or times to take part, or having personal or technological impracticalities such as a lack of childcare or no access to a laptop/internet, which impede one’s ability to take part in research. Our hypothesised barrier of ‘transportation problems’ did arise within the subtheme ‘travel’, however, this item was not one of the highest ranked. It is within the other subthemes that highest-ranked barriers are present, such as not having internet access or necessary equipment, not having time to take part or being restricted by the participation dates, and the research location not being accessible; specifically, if the research is in a ‘…public place/unknown environment’. Directly mapping onto this barrier, ‘improved accessibility’ was the largest facilitator theme, with subthemes ‘practical accessibility’, ‘timings’, ‘participation options’ and ‘communication/advertisement’. This theme encompasses the practical aspects of accessibility in addition to having accessible timings of and accessible/alternative options for taking part. ‘Extra support’ and ‘having both online and in-person option(s) available’ were top-ranked facilitators within these subthemes with ‘flexibility in when to participate’ and the length of time spent participating mentioned as additional considerations.

The third largest barrier theme was ‘chronic symptoms and comorbidities’ with the subthemes ‘fatigue’, ‘psychological symptoms’ and ‘physical symptoms’. This theme is characterised by physical and mental health conditions that are either associated with a chronic pain condition, or experienced concurrently, and which negatively impact participation ability. Within the ‘fatigue’ subtheme, one of the more common barriers concerned the impact that taking part in research can have; a ‘worry of future fatigue (as a consequence of taking part)’, which was echoed within the ‘physical symptoms’ subtheme as a ‘fear of increase in pain (in other areas)’. These items highlight the fear/worry about the potential physical cost involved for people with chronic pain when considering taking part in research, with participants reporting expectations of pain and fatigue both during and after research participation. Linking to this barrier theme the second largest facilitator theme ‘positive impact of participation’ arose, with subthemes regarding positive ‘impact on day’ and ‘impact after’. Highly ranked items concerning impact on the day were around ‘improvement in pain’ and ‘knowing there are others who experience the (same) issues’, and ‘feeling a part of the community’. Regarding positive impact after participating, facilitator items included knowing that ‘research could facilitate potential new methods’, ‘knowing if research can have long-term benefits’, ‘more information about the benefits of the research’ and ‘get(ting) feedback about the research outputs’ in an accessible manner.

‘Lack of information’ was the fourth largest barrier theme identified and consisted of subthemes ‘study details’ and ‘recruitment/study advertisement’ and was characterised by a lack of information regarding the study details and what taking part would involve, or a lack of awareness of studies due to the methods used for study advertisement. Within the ‘study details’ subtheme, the main concern was around unknown expectations when taking part in research, along with a concern regarding a ‘lack of understanding about non-pharmaceutical treatments’ from both patient and professional perspectives. ‘Little or no awareness of research happening/lack of advertising of research’ was a top-ranked barrier within the ‘recruitment/study advertisement’ subtheme, along with ‘not knowing about the research because it is not often spoken about’ and ‘not being aware that research takes place/not being mentioned in doctors’ surgery’. Mapping directly onto this barrier theme is the third largest facilitator theme ‘detailed and accessible information’, with subthemes ‘eligibility’ and ‘research’. This theme related to having increased information about the research and what taking part would involve, and the ‘eligibility’ subtheme concerns making sure participants are aware of which diagnoses are relevant to the project and possibly knowing others who have taken part. The ‘research’ subtheme consisted of items related to having ‘very detailed information about what to expect’ which should consist of ‘knowing research aims’ and research methodology, such as having ‘clarity about group or one-to-one sessions’ in addition to ‘more information about the research and benefits’ which all arose as highly ranked items within this subtheme.

Within the fifth barrier theme ‘lack of motivation’, responses described the lack of motivation to take part in research, either because this was not a priority for respondents, or because there were not enough incentives or compensation for their time. ‘Lack of priority’ arose as a major barrier in relation to research participation, with statements such as taking part ‘not (being) a priority because of other pain’ and ‘caring responsibilities being the focus’. This barrier theme also highlights the effect that a ‘lack of incentives to take part’ can have on people’s motivation to offer their time and resources to research, especially when doing so is perceived as having the potential to be detrimental to their physical and mental well-being. Within the fourth largest facilitator theme ‘increased motivation’, potential incentives for participation encompassed top-ranked items such as ‘financial compensation for your time for participation’, ‘incentive for taking part (financial/other)’ and ‘improvement in pain’.

Within the sixth barrier theme that was identified from the data; ‘cultural barriers/individual differences’, there were no top-ranking items nor subthemes. This theme was characterised by differences in cultural views of chronic pain, such as differences in management/labelling of chronic pain, or individual differences such as sensory needs or learning disabilities. The barrier items within this theme can be linked to the seventh and final barrier theme ‘self-identification/eligibility’ which is described as a lack of understanding regarding research participation eligibility criteria, lacking an official chronic pain condition (and being unsure if this affects eligibility), and general denial about having chronic pain. Here, the item ‘not feeling like you can take part if you don't have a diagnosis’ arises as a topmost barrier.

The fifth and final facilitator theme was ‘safe space’ which focused on a holistic view of research and can be described as having an environment for research that includes approachable researchers, possibly those with lived experience of chronic pain, the possibility of having an accompanying companion during participation and taking part in a space that is not in a research or clinical setting. Top-ranked items mentioned within this theme were having ‘approachable researchers’ and ‘smaller focus groups (being) more comfortable/discuss in safe space/anonymity’. Other more holistic approaches raised included having caring researchers who are understanding and who are willing and able to discuss participant needs prior to participation.

## Phase 2 methods

### Preregistration

The preregistration for this study can be found on OSF at the following DOI: https://doi.org/10.17605/OSF.IO/37SNZ. Slight deviations from the preregistration consist of the following:

- Phase 2 planned to ask participants to confirm the themes reached through thematic analysis in phase 1, however, this was deemed impractical without adequate training on thematic analysis for participants.
- Phase 2 stated the inclusion of data where only 100% of the survey was completed, however after phase 1 highlighted that fatigue can act as a barrier to take part in research, this was revised to acceptance of data if 100% of either the barriers or facilitators questions were completed.

### Participants

103 participants with chronic pain conditions (89% female, 10% male, 1% prefer not to say; age=20–80 years, M=46.6 years) and based within the UK, responded to the phase 2 online survey. The sample size was decided to be larger than that included within phase 1, aiming for over 100 participants, to try and gain a wider understanding of barriers and facilitators among people living with chronic pain across the UK. There is no standard method for calculating the sample size for a Delphi interview technique (see "Data Collection" for details) following the use of the NGT, however, previous studies using both techniques have reported samples between 9 and 141 participants.^30^ Therefore, the sample size aim of over 100 for the present study was to fit within the samples sizes from previous research whist aiming to get a large enough sample to increase the variety of experiences gathered from people living with chronic pain in the UK. Demographic information for these participants can be seen in table 2. A list of the chronic pain conditions within this participant sample can be seen in online supplemental table S4. All participants gave informed consent before taking part in the survey.

**Table 2:** Ethnicity and highest education level achieved for phase 2 participants

Ethnicity		Education level	
White	96%	College (A-level equivalent)	30%
Mixed/multiple ethnic groups	2%	Undergraduate degree	29%
Black, Black British, Caribbean or African	1%	Master’s degree	19%
Other ethnic group	1%	Secondary school	15%
		Postgraduate PhD or MD	5%
		Primary school	2%

### Patient and public involvement

Patients and the public were not involved in the design, or conduct, or reporting, or dissemination plans of our research. Public chronic pain groups were contacted to help with the advertisement of the research.

### Data collection

After running the focus groups, the Delphi technique was used through creating a questionnaire containing all themes regarding both barriers and facilitators to take part in non-pharmacological chronic pain research. This study employed one of the most common approaches to the Delphi technique^21^ through using data from phase 1’s qualitative approach to form the questionnaire items for the subsequent quantitative data generation within phase 2. This phase 2 questionnaire was distributed online and through chronic pain support groups to facilitate assessment of wider agreement or disagreement with the themes identified from the focus groups. Participants were asked to assess their personal perspective regarding each theme and respond with their level of agreement or disagreement. The questionnaire was created using Qualtrics (Qualtrics, Provo, Utah, USA), and included Likert scales ranging from −3 indicating strong disagreement to +3 indicating strong agreement, and 0 indicating a neutral opinion of the theme. The seven themes identified as barriers in phase 1 were presented, along with the five themes identified as facilitators. Each theme was stated using a title, followed by a short definition, which was created using the items listed within the theme from the focus groups. For example, the theme ‘distrust’ was followed by ‘…this is described as having distrust of the level of anonymity and confidentiality or having a distrust of medical or research professionals’. Researchers KH and CP collectively decided on the statements to give for each theme, making sure to include as much detail from the items as possible without making the statements too long for participants to read. After all barriers themes were presented, participants were asked if there were any other barriers that they experienced that were not mentioned within the themes presented. The same was asked following presentation of all facilitator themes. The questionnaire, including all themes and definitions, can be seen in online supplemental appendix A.

Phase 2 data and analysis code can be seen on the following OSF page: https://osf.io/8y7rz/. Date of birth and qualitative data have been removed to prevent participant identification.

### Data analysis

Data were exported from Qualtrics and responses ranging from +1 to +3 were classified as indicative of agreement with the theme and were coded with an ‘A’, while scores of −1 to −3 were indicative of disagreement with the theme and were coded with a ‘D’, and finally, scores of 0 were indicative of a neutral stance regarding the theme and were coded with an ‘N’. Percentages were calculated for overall agreement, disagreement and neutral responses, in line with the preregistration. Further exploratory analyses were conducted regarding the level of agreement and disagreement within each theme. The free text sections included within the questionnaire asked if the participant was aware of any additional barriers or facilitators that were not included within the themes mentioned, allowing for further exploratory qualitative analyses. This qualitative analysis of phase 2 data involved assessing if any items fit within an existing theme, or within a potential new theme/subtheme.

## Phase 2 results

**Figure 1:**
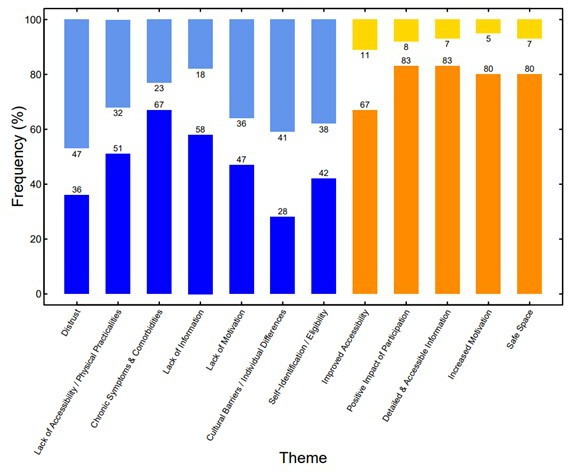
Percentage agreement (bottom bars) and percentage disagreement (top bars) for each barrier and facilitator theme. Blue shaded bars represent barriers while orange shaded bars represent facilitators. The difference between the agreement and disagreement percentages gives the percentage of participants who gave a neutral response regarding their opinion of a theme.

Percentage agreement, disagreement and neutral responses relating to each barrier and facilitator theme were calculated across all participants and can be seen in figure 1.

Regarding agreement, the barrier theme with the highest percentage was ‘chronic symptoms and comorbidities’, closely followed by ‘lack of information’, with ‘cultural barriers/individual differences’ getting the lowest level of agreement among the barrier themes. All facilitator themes achieved a similarly high level of agreement.

For disagreement, all themes had relatively low levels, apart from the barrier theme ‘distrust’, where almost half of participants disagreed with this theme. This was followed by ‘cultural barriers/individual differences’, ‘self-identification/eligibility’ and ‘lack of motivation’ having around 40% of participants disagreeing with these barrier themes. ‘Distrust’ and ‘cultural barriers/individual differences’ were the only two themes that showed more disagreement than agreement.

Exploratory analyses (online supplemental figure S5) showed a greater degree of variation (in terms of agreement/disagreement) with the phase 1 barrier themes, whereas there was moderate to strong agreement from phase 2 respondents with the facilitator themes identified in phase 1.

### Analysis of additional barriers and facilitators

Within the survey, participants were asked to list any additional barriers/facilitators that they experienced that differed from the ones mentioned. 22 participants mentioned additional barriers and 5 mentioned additional facilitators. Some participants mentioned more than one item within their responses. Following data familiarisation, two researchers (KH and DB) independently assessed whether these items comprised new themes or fit within an existing theme. Agreement between the two researchers was high for barriers items; barrier theme allocation percentage agreement: 80%,^26 27^ Cohen’s kappa: 0.789 (moderate agreement^28^; substantial agreement^29^), and for facilitator items: facilitator theme allocation percentage agreement: 84.61%,^26 27^ Cohen’s kappa: 0.634 (moderate agreement^28^; substantial agreement^29^). Both researchers agreed on the final allocation of items to existing themes.

Of the additional barrier responses, eight could be placed within the ‘lack of information’ theme, six within ‘chronic pain symptoms and comorbidities’, 5 within ‘self-identification/eligibility’, three within ‘distrust’ and four within ‘lack of accessibility/physical practicalities’. Importantly, within two responses, the item ‘rarity of diagnosis’ was mentioned, which while fitting under the theme ‘self-identification/eligibility’ could create a new subtheme pertaining to how common the diagnosis is, with less research likely available for those with rarer diagnoses.

Regarding facilitator responses, no new themes were identified, as four could be placed with the theme ‘improved accessibility’ and one within ‘positive impact of participation’.

## Discussion

This study assessed barriers and facilitators to non-pharmaceutical research participation for people with chronic pain. Although our hypothesised barriers ‘transportation problems’ and ‘a lack of understanding of non-pharmaceutical treatments for chronic pain’ were present, they were not found to be the largest. Instead ‘distrust’ was the largest barrier theme identified and ‘improved accessibility’ was the largest facilitator theme. Overall, more people in phase 2 agreed than disagreed with each theme from phase 1, with two key exceptions: barrier themes ‘distrust’ and ‘cultural barriers/individual differences’. Exploratory analyses revealed larger variation within opinions for barrier themes while showing overall agreement across facilitator themes.

Phase 1 analysis found the topmost barrier to participation in non-pharmaceutical research to be a ‘lack of understanding about chronic pain’ under the largest barrier theme ‘distrust’. This distrust of researchers’ and medical professionals’ understanding of the lived experience of chronic pain is unsurprising considering UK medical schools dedicate a median of only 13 hours to teaching pain medicine over 5 years, and only 4% have a dedicated pain science module.^31^ Interestingly, phase 2 analyses highlighted that almost 50% of the wider sample disagreed with experiencing distrust, however, this is possibly due to a self-selection bias, as individuals who experience distrust of research are less likely to have taken part in our study. One further caveat is that phase 1 participants assessed barriers and facilitators for themselves and others living with chronic pain, whereas phase 2 participants only gave personal reflections. This difference likely explains the pattern of results seen, with phase 1 participants speaking to a distrust that others might have of researchers’ and medical professionals’ understanding of chronic pain.

Our finding that a lack of time is a barrier to participation was supported by the attrition data for phase 1, whereby 19% of participants recruited were unable to attend their focus group or a subsequent session. Although restrictive participation dates may not appear to be a barrier specific to those with chronic pain, the lack of control and unpredictability of chronic pain symptoms often results in participants needing to cancel participation at short notice,^32^ therefore, this population group is likely disproportionately affected by this barrier. Further evidence for this barrier arose within our third largest barrier theme ‘chronic symptoms and comorbidities’, with phase 2 analysis finding this theme to have the highest overall agreement of barrier themes, with 67% of participants agreeing that it can prevent non-pharmaceutical research participation. One of the most pressing barriers to participation, and arguably the easiest to fix, arose within the ‘lack of information’ theme; people simply do not know research is happening. Participants mentioned that improving awareness of participation opportunities could come from advertising within community and medical spaces, such as the National Health Service, general practitioner surgeries and places of faith/worship. Participants highlighted that research advertisement should come through trusted routes, rather than unknown sources. The importance of accessible advertising, such as different formats (visual/audio) and different languages, was also emphasised. The facilitator theme ‘detailed and accessible information’ received the highest level of agreement within phase 2, clearly reinforcing this need for inclusive research advertisement through trusted sources.

The barrier theme ‘cultural barriers/individual differences’ received more disagreement than agreement within phase 2, likely due to the overrepresentation of white female participants in both samples. Nevertheless, a main concern within this theme related to learning disabilities and sensory issues, which is important to consider since research has suggested links between chronic pain and neurodivergent conditions which often consist of sensory issues and/or learning disabilities. Research has highlighted links between joint hypermobility, which underlies several chronic pain conditions, and conditions such as autism and attention defecit hyperactivity disorder (ADHD).^33^ Additionally, in a large, self-selected community population, fibromyalgia symptoms were found to be significantly associated with autistic traits.^34^ Therefore, despite research connecting neurodivergence and chronic pain being somewhat in its infancy, and there being no explicit mention of autism or ADHD within the focus group items presented within this study (only a mention of ‘sensory issues’), it is important to design research studies which encompass learning and sensory accommodations to encourage participation from everyone with chronic pain, including accommodating potential neurodivergent conditions which may be comorbid. One barrier raised related to culturally mediated distinctions between experiencing chronic pain as a natural outcome of ageing or regarding chronic pain as pathological. However, since there were few items relating to this within our sample, it is important to consider conclusions based on this potential dichotomy as tentative and in need of replication in samples from more ethnically diverse populations. The barrier theme ‘self-identification/eligibility’ also raised concerns over knowing if you could take part without a specific diagnosis of a chronic pain condition, which is likely a further barrier for those from ethnically diverse backgrounds where diagnoses may show different prevalence rates.^35^ Findings within this theme underscore the intersectional barriers that individuals with chronic pain from diverse backgrounds face when considering taking part in research, despite the relatively homogenous nature of our sample. A recommendation to facilitate participation from diverse backgrounds is to be clear about what constitutes eligibility, showing a defined symptom profile (such as having lasting or reoccurring pain for more than 3 months^1 2^), rather than a list of diagnoses.

Considering the impact that research participation can have on individuals with chronic pain—from physical impact, time commitments and psychological impacts—compensation and additional incentives must be given to participants. The facilitator theme ‘increased motivation’ demonstrates that compensation can come from sources such as financial remuneration, pain improvement, travel reimbursement and altruistic motivations. The facilitator theme ‘positive impact of participation’ highlights that feeling part of a community can also have long-term benefits. However, there have been issues with compensation for research becoming an incentive for ‘imposter participants’; those who are motivated to take part in research for financial gain and represent themselves inauthentically. For example, a recent study commented on large language models being used by imposter participants to give detailed qualitative responses during focus group research projects.^36^ Therefore, it is important to consider trusted routes for research advertisement to try and avoid such imposters diluting clinical data. Another facilitator suggested including researchers with lived experience of chronic pain. Although this may not always be possible to achieve within existing research groups, methods such as patient and public involvement in research, research coproduction, or the inclusion of patient experts within the research team can ensure that those with lived experience are included in the research process. Previous research assessing the barriers that patients with persistent pain face when acting as patient advocates found a lack of financial compensation, and inflexible deadlines existed as barriers,^37^ which is directly supported by barrier items identified here relating to inflexible time and a lack of compensation and maps directly to the facilitators found regarding a need for incentives, compensation and flexibility within the research environment. This highlights that these concerns need to be considered when engaging with samples with chronic pain, either as participants or advocates.

There were some methodological limitations present in the current study, such as the use of online measures to collect both focus group and questionnaire data, which despite being used to try and encourage participation from as many people as possible, would have prevented people without internet access from taking part. Additionally, despite phase 2 having a much larger sample size than phase 1, the aim of this phase was to gather data from a wider and more diverse sample than that collected in phase 1. Unfortunately, the data collected in both phases were almost exclusively from people of a white ethnic background, which means that generalising these findings to people from other ethnic backgrounds might be difficult and highlights the need for more research into barriers and facilitators to participation from minoritised or marginalised ethnic groups within the UK. It is possible that the lack of inclusion of a diverse range of interested parties in the design of the study could have contributed to this over-representation of white women within the sample, therefore, it is highly recommended that future research includes contributions from, and collaboration with, people from a wider range of communities and ethnic backgrounds in the design and advertisement of potential projects. One final methodological concern relates to the statements used in the phase 2 questionnaire. These statements were derived from the items generated within phase 1, but since so many items were generated, it was not possible to list all of them for the purposes of the questionnaire. Therefore, it is possible that the levels of agreement and disagreement with the themes could have differed if all items were presented to phase 2 participants. Due to time constraints, this was not the approach used, but it is important to note that phase 2 data relates directly to the statement definitions of the themes (which can be seen in online supplemental appendix A), rather than the complete lists provided in online supplemental tables S2,S3.

Despite these limitations, the data nonetheless provide several clear recommendations for improving non-pharmaceutical chronic pain research participation. When creating a research group, having approachable researchers and including people with lived experience is vital to address the distrust and fear that participants report experiencing when considering taking part. The environment in which research will be conducted must be accessible, comfortable and ideally in a non-academic/non-clinical setting. Advertisement of participation opportunities should be in community spaces, through trusted communication routes and must include detailed and accessible information about the research aims and what is involved in taking part. Eligibility must detail specific symptom profiles to encourage participation from all ethnic groups, and researchers must be contactable to discuss accessibility requirements prior to participation. If participation could potentially increase pain, this must be made clear from the outset. During research sessions, accessible participation options including the use of public internet/computers, flexible timings/data input formats, and translation services must be given. Having the possibility of a friend or carer attending the research appointment should be accounted for and breaks must be offered to reduce fatigue. After participation, participants must be adequately compensated for their time and travel, and the longer-term benefits or implications of the research must be made clear. Finally, a lay summary of research findings should be offered to participants, to ensure they are aware of the impact that their participation has had on research outputs.

## Conclusions

This project addresses an apparent gap in our understanding of what prevents people with chronic pain conditions from taking part in non-pharmaceutical research. Across two distinct samples of participants, facilitators have been identified, consolidated and are recommended to be implemented in all applicable chronic pain research settings. Barriers and facilitators identified here are likely generalisable to both pharmaceutical and non-pharmaceutical projects, as there were limited items specifically related to non-pharmaceutical research identified during the focus groups. Considering these barriers and facilitators when developing research programmes for chronic pain treatment is likely to encourage the involvement of individuals with chronic pain throughout the research process, and thus the likelihood of designing effective non-pharmaceutical therapies should be greatly increased.

## supplementary material

10.1136/bmjopen-2024-089676 online supplemental file 1

10.1136/bmjopen-2024-089676 online supplemental file 2

## Data Availability

Data are available in a public, open access repository.
