# Supplementary material for: Understanding barriers and facilitators to non-pharmaceutical chronic pain research engagement among people living with chronic pain in the UK: a two-phase mixed-methods approach
Source: BMJ Open. 2024 Dec 22;14(12):e089676. doi: 10.1136/bmjopen-2024-089676 (PMC11751962; doi:10.1136/bmjopen-2024-089676)
Supplement: online supplemental file 1 [file bmjopen-14-12-s001.docx]

**Appendix A:**

**Phase 2 Questionnaire:**

**Barriers and Facilitators to Taking Part In Non-Pharmaceutical Based Chronic Pain Research**
 Thank you for expressing interest in taking part in our study about the barriers and facilitators to taking part in non-pharmaceutical based (drug-free) chronic pain research!

 This is the second phase to a project looking at what makes it difficult (barriers) and what could make it easier (facilitators) for people with chronic pain to take part in research studies.

 Phase 1 consisted of running 7 focus groups, where people with chronic pain conditions were asked to give their opinions of the barriers and facilitators that exist towards taking part in research studies, and from the items given in these groups, we have identified several overarching themes for barriers and facilitators.

 In this phase 2 of the study, we are wanting to see the level of agreement / disagreement with these themes for a wider sample of people with chronic pain conditions. 

 If you have any questions please contact principle investigator Dr Catherine Preston (catherine.preston@york.ac.uk) or PhD researcher Kirralise Hansford (kirralise.hansford@york.ac.uk).

Please complete a few consent questions below before taking part in the study:

 I have read and understood the information sheet entitled ‘Barriers and Facilitators to Participation in Non-Pharmaceutical Based Chronic Pain Research’, and I have asked any questions I had via email and, if applicable, I have had satisfactory answers to any questions:

- Yes (1)
- No (2)

I understand that I am free to withdraw from the study:
- At any time
- Without having to give a reason

- Yes (1)
- No (2)

If I have any questions or concerns about the research, I know I can contact Kirralise Hansford via email - kirralise.hansford@york.ac.uk

- Yes (1)
- No (2)

I accept the terms and conditions of this study and agree to take part.

- Yes (1)
- No (2)

| Page Break |  |
| --- | --- |

First, we please ask you to complete the following questions about yourself, so that we can collect data on the representation of the population within our sample:

| 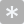 |
| --- |

Please enter your date of birth (dd/mm/yyyy):

________________________________________________________________

Please detail the location where you primarily live:

- Within the UK (1)
- Outside of the UK (2)

Please select your sex:

- Male (1)
- Female (2)
- Prefer not say (3)

Please enter the chronic pain condition/s you have been diagnosed with:

________________________________________________________________

Please select your highest education level:

- Primary School (1)
- Secondary School (GCSE or equivalent) (2)
- College level (A-level / BTEC or equivalent) (3)
- Undergraduate Degree (Bachelor's) (4)
- Postgraduate Degree (Masters) (5)
- Postgraduate Degree (PhD / MD) (6)

Please select your ethnicity:

- Asian or Asian British (1)
- Black, Black British, Caribbean or African (2)
- Mixed or Multiple Ethnic Groups (3)
- White (4)
- Other Ethnic Group (5)

| Page Break |  |
| --- | --- |

Barriers On the coming pages you will be presented with different themes for barriers and we ask you to rate your level of agreement as to how much this applies to you on a scale from -3 (strongly disagree) to +3 (strongly agree).

If you agree that the each theme acts as a barrier for you taking part in chronic pain research, then please give a score between 1 and 3 based on your level of agreement.

However, if you disagree, then please give a score between -1 and -3 based on your level of disagreement.

If you are unsure then please give a rating of 0.

There are a total of 7 barriers, each presented on a new page.

| Page Break |  |
| --- | --- |

For the first barrier, "Distrust", this is described as having distrust of the level of anonymity and confidentiality, or having a distrust of medical or research professionals.

Please indicate your level of agreement / disagreement with "Distrust" acting as a barrier for you taking part in non-pharmaceutical based chronic pain research on the scale below. 

 -3 = "Strongly disagree"
 -2 = "Disagree"
 -1 = "Somewhat disagree"
  0 = "Neither agree nor disagree"
+1 = "Somewhat agree"
+2 = "Agree"
+3 = "Strongly agree"

|  | Please give your rating: |
| --- | --- |

|  | -3 | -2 | -1 | 0 | 1 | 2 | 3 |
| --- | --- | --- | --- | --- | --- | --- | --- |

| Distrust (Including: Distrust of anonymity / confidentiality; Distrust of the impact of the research; Distrust of medical / research professionals and settings) () | 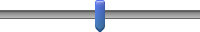 |
| --- | --- |

| Page Break |  |
| --- | --- |

The second barrier, "Lack of Accessibility / Physical Practicalities", is described as having a lack of accessibility or physical practicalities within the research such as inaccessible travel options or times to take part, or having personal or technological impracticalities such as a lack of childcare or a laptop/internet. 

 Please indicate your level of agreement / disagreement with "Lack of Accessibility / Physical Practicalities" acting as a barrier for you taking part in non-pharmaceutical based chronic pain research on the scale below.

  -3 = "Strongly disagree" 
 -2 = "Disagree" 
 -1 = "Somewhat disagree"
  0 = "Neither agree nor disagree" 
+1 = "Somewhat agree"
+2 = "Agree"
+3 = "Strongly agree"

|  | Please give your rating: |
| --- | --- |

|  | -3 | -2 | -1 | 0 | 1 | 2 | 3 |
| --- | --- | --- | --- | --- | --- | --- | --- |

| Lack of Accessibility / Physical Practicalities (Including; lack of accessible times to take part; Lack of accessible travel; Lack of childcare or technology to take part) () | 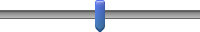 |
| --- | --- |

| Page Break |  |
| --- | --- |

The third barrier, "Chronic Symptoms & Comorbidities", is described as chronic pain symptoms and comorbidities such as fatigue, psychological and / or physical symptoms. 

Please indicate your level of agreement / disagreement with "Chronic Symptoms & Comorbidities" acting as a barrier for you taking part in non-pharmaceutical based chronic pain research on the scale below.

 -3 = "Strongly disagree"
 -2 = "Disagree"
 -1 = "Somewhat disagree"
  0 = "Neither agree nor disagree"
+1 = "Somewhat agree"
+2 = "Agree"
+3 = "Strongly agree"

|  | Please give your rating: |
| --- | --- |

|  | -3 | -2 | -1 | 0 | 1 | 2 | 3 |
| --- | --- | --- | --- | --- | --- | --- | --- |

| Chronic Symptoms & Comorbidities (Including; Fatigue; Psychological symptoms; Physical symptoms) () | 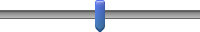 |
| --- | --- |

| Page Break |  |
| --- | --- |

The fourth barrier, "Lack of Information", is described as having a lack of information about the study details and what taking part would involve, or there being a lack of advertisement about the study.

 Please indicate your level of agreement / disagreement with "Lack of Information" acting as a barrier for you taking part in non-pharmaceutical based chronic pain research on the scale below. 

  -3 = "Strongly disagree"
  -2 = "Disagree"
  -1 = "Somewhat disagree"
   0 = "Neither agree nor disagree"
 +1 = "Somewhat agree"
 +2 = "Agree"
 +3 = "Strongly agree"

|  | Please give your rating: |
| --- | --- |

|  | -3 | -2 | -1 | 0 | 1 | 2 | 3 |
| --- | --- | --- | --- | --- | --- | --- | --- |

| Lack of Information (Including; Lack of information about what is involved; Lack of knowledge of advertisement of the research study) () | 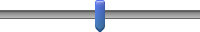 |
| --- | --- |

| Page Break |  |
| --- | --- |

The fifth barrier, "Lack of Motivation", is described as having a lack of motivation to take part, either from taking part being a lack of priority for you, or that there not being enough incentives or compensation for your time. 

 Please indicate your level of agreement / disagreement with "Lack of Motivation" acting as a barrier for you taking part in non-pharmaceutical based chronic pain research on the scale below.

  -3 = "Strongly disagree"
  -2 = "Disagree"
  -1 = "Somewhat disagree"
   0 = "Neither agree nor disagree"
 +1 = "Somewhat agree"
 +2 = "Agree"
 +3 = "Strongly agree"

|  | Please give your rating: |
| --- | --- |

|  | -3 | -2 | -1 | 0 | 1 | 2 | 3 |
| --- | --- | --- | --- | --- | --- | --- | --- |

| Lack of Motivation (Including; Lack of priority; Lack of incentives / compensation) () | 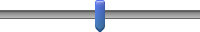 |
| --- | --- |

| Page Break |  |
| --- | --- |

The sixth barrier, "Cultural Barriers / Individual Differences", is described as differences in cultural views of chronic pain, such as differences in management / labelling of chronic pain, or individual differences such as sensory needs or learning disabilities.

 Please indicate your level of agreement / disagreement with "Cultural Barriers / Individual Differences" acting as a barrier for you taking part in non-pharmaceutical based chronic pain research on the scale below. 

  -3 = "Strongly disagree"
  -2 = "Disagree"
  -1 = "Somewhat disagree"
   0 = "Neither agree nor disagree"
 +1 = "Somewhat agree"
 +2 = "Agree"
 +3 = "Strongly agree"

|  | Please give your rating: |
| --- | --- |

|  | -3 | -2 | -1 | 0 | 1 | 2 | 3 |
| --- | --- | --- | --- | --- | --- | --- | --- |

| Cultural Barriers / Individual Differences (Including: Different cultural views on chronic pain; Sensory issues; Learning disabilities) () | 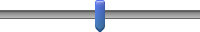 |
| --- | --- |

| Page Break |  |
| --- | --- |

The seventh and final barrier, "Self Identification / Eligibility", is described as a lack of understanding if you are eligible for the research study, whether you lack a diagnosis for a chronic pain condition, or general denial.  

 Please indicate your level of agreement / disagreement with "Self Identification / Eligibility" acting as a barrier for you taking part in non-pharmaceutical based chronic pain research on the scale below.

  -3 = "Strongly disagree"
  -2 = "Disagree"
  -1 = "Somewhat disagree"
   0 = "Neither agree nor disagree"
 +1 = "Somewhat agree"
 +2 = "Agree"
 +3 = "Strongly agree"

|  | Please give your rating: |
| --- | --- |

|  | -3 | -2 | -1 | 0 | 1 | 2 | 3 |
| --- | --- | --- | --- | --- | --- | --- | --- |

| Self Identification / Eligibility (Including: Lack of diagnosis; Understanding eligibility) () | 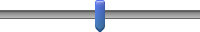 |
| --- | --- |

| Page Break |  |
| --- | --- |

Are there any other barriers (things that would make it harder for you to take part in research) that you think are not already mentioned within the themes you just saw?

________________________________________________________________

________________________________________________________________

________________________________________________________________

________________________________________________________________

________________________________________________________________

| Page Break |  |
| --- | --- |

Next, on the coming pages you will be presented with different themes for facilitators and we ask you to again rate your level of agreement as to how much this applies to you on a scale from -3 (strongly disagree) to +3 (strongly agree).

If you agree that the each theme acts as a facilitator (thing that would make it easier) for you taking part in chronic pain research, then please give a score between 1 and 3 based on your level of agreement.

However, if you disagree, then please give a score between -1 and -3 based on your level of disagreement.

If you are unsure then please give a rating of 0.

There are a total of 5 facilitators, each presented on a new page.

| Page Break |  |
| --- | --- |

The first facilitator, "Accessibility", is described as having accessible research such as including practical aspect of accessibility and accessible timings of research, or accessible options of how to take part. 

 Please indicate your level of agreement / disagreement with "Accessibility" acting as a facilitator for you taking part in non-pharmaceutical based chronic pain research on the scale below.

  -3 = "Strongly disagree"
  -2 = "Disagree"
  -1 = "Somewhat disagree"
   0 = "Neither agree nor disagree"
 +1 = "Somewhat agree"
 +2 = "Agree"
 +3 = "Strongly agree"

|  | Please give your rating: |
| --- | --- |

|  | -3 | -2 | -1 | 0 | 1 | 2 | 3 |
| --- | --- | --- | --- | --- | --- | --- | --- |

| Accessibility (Including: Practical accessibility and timings; Differing participation options; Accessible communication & Advertisement) () | 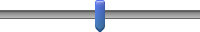 |
| --- | --- |

| Page Break |  |
| --- | --- |

The second facilitator, "The Positive Impact of Participation", is described as taking part in research and it having a positive impact on you on that day and after through acknowledgement, community, and potential pain relief. 

 Please indicate your level of agreement / disagreement with "The Positive Impact of Participation" acting as a facilitator for you taking part in non-pharmaceutical based chronic pain research on the scale below.

  -3 = "Strongly disagree"
  -2 = "Disagree"
  -1 = "Somewhat disagree"
   0 = "Neither agree nor disagree"
 +1 = "Somewhat agree"
 +2 = "Agree"
 +3 = "Strongly agree"

|  | Please give your rating: |
| --- | --- |

|  | -3 | -2 | -1 | 0 | 1 | 2 | 3 |
| --- | --- | --- | --- | --- | --- | --- | --- |

| The Positive Impact of Participation (Including: Impacting you on the day and after through acknowledgement, community, and potential pain relief) () | 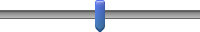 |
| --- | --- |

| Page Break |  |
| --- | --- |

The third facilitator, "Increased Information", is described as having increased information such as information about the research and what taking part would involve, and information on who is eligible to take part.

 Please indicate your level of agreement / disagreement with "Increased Information" acting as a facilitator for you taking part in non-pharmaceutical based chronic pain research on the scale below. 

  -3 = "Strongly disagree"
  -2 = "Disagree"
  -1 = "Somewhat disagree"
   0 = "Neither agree nor disagree"
 +1 = "Somewhat agree"
 +2 = "Agree"
 +3 = "Strongly agree"

|  | Please give your rating: |
| --- | --- |

|  | -3 | -2 | -1 | 0 | 1 | 2 | 3 |
| --- | --- | --- | --- | --- | --- | --- | --- |

| Increased Information (Including Information about: The research project; Who is eligible to take part) () | 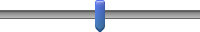 |
| --- | --- |

| Page Break |  |
| --- | --- |

The fourth facilitator, "Increased Motivation to Take Part", is described as having increased motivation to take part such as having interesting research and having compensation and incentives to take part, and the possibility for improvement in pain.  

 Please indicate your level of agreement / disagreement with "Increased Motivation to Take Part" acting as a facilitator for you taking part in non-pharmaceutical based chronic pain research on the scale below.

  -3 = "Strongly disagree"
  -2 = "Disagree"
  -1 = "Somewhat disagree"
   0 = "Neither agree nor disagree"
 +1 = "Somewhat agree"
 +2 = "Agree"
 +3 = "Strongly agree"

|  | Please give your rating: |
| --- | --- |

|  | -3 | -2 | -1 | 0 | 1 | 2 | 3 |
| --- | --- | --- | --- | --- | --- | --- | --- |

| Increased Motivation to Take Part (Including: Having interesting research; Compensation & Incentives; Improvement in pain) () | 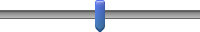 |
| --- | --- |

| Page Break |  |
| --- | --- |

The fifth and final facilitator, "Safe Space", is described as having a safe space for research such as having approachable researchers, possibly those with lived experience of chronic pain, or being able to have a companion with you whilst taking part, or taking part in a space that wasn't in a research or clinical setting. 

 Please indicate your level of agreement / disagreement with "Safe Space" acting as a facilitator for you taking part in non-pharmaceutical based chronic pain research on the scale below.

  -3 = "Strongly disagree"
  -2 = "Disagree"
  -1 = "Somewhat disagree"
   0 = "Neither agree nor disagree"
 +1 = "Somewhat agree"
 +2 = "Agree"
 +3 = "Strongly agree"

|  | Please give your rating: |
| --- | --- |

|  | -3 | -2 | -1 | 0 | 1 | 2 | 3 |
| --- | --- | --- | --- | --- | --- | --- | --- |

| Safe Space (Including: Having approachable researchers, possibly with lived experience; Having a companion with you; Non-clinical / non-academic setting) () | 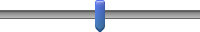 |
| --- | --- |

| Page Break |  |
| --- | --- |

Are there any other facilitators (things that would make it easier for you to take part in research) that you think are not already mentioned within the themes you just saw?

________________________________________________________________

________________________________________________________________

________________________________________________________________

________________________________________________________________

________________________________________________________________
