# Supplementary material for: Understanding barriers and facilitators to non-pharmaceutical chronic pain research engagement among people living with chronic pain in the UK: a two-phase mixed-methods approach
Source: BMJ Open. 2024 Dec 22;14(12):e089676. doi: 10.1136/bmjopen-2024-089676 (PMC11751962; doi:10.1136/bmjopen-2024-089676)
Supplement: online supplemental file 2 [file bmjopen-14-12-s002.pdf]

**Supplementary:**

**S1 Table of chronic pain conditions prevalent within phase 1**

*Table S1.* Prevalence of chronic pain condition within focus group participant sample. Note: Several participants had more than one chronic pain condition, therefore the total of the percentages does not equal the total number of participants.

| Chronic Pain Condition | Prevalence within Participant Sample |
|---|---|
| Chronic Back Pain | 28% |
| Rheumatoid Arthritis | 24% |
| Fibromyalgia | 17% |
| Osteoarthritis | 14% |
| Neck Pain | 10% |
| Juvenile Idiopathic Arthritis (JIA) | 7% |
| Chronic Regional Pain | 3% |
| Spinal Stenosis | 3% |
| Ganglion Cysts | 3% |
| IBS | 3% |
| Gynaecological Pains | 3% |
| Crohn’s Disease | 3% |
| Facial Pain | 3% |
| Ehlers Danlos Syndrome | 3% |
| Hip Labral Tear | 3% |
| Psoriatic Arthritis | 3% |
| Mixed Connective Tissue Disease | 3% |
| Temporomandibular Joint Disorder (TMJ) | 3% |

**S2 Table of Barriers from Phase 1**

*Table S2.* Themes and subthemes for barrier items, with the topmost facilitators from a focus group indicated with a superscript "1st", second topmost facilitators indicated with a "2nd", and third topmost facilitators indicated with a "3rd". Items which received votes but did not rank within the top 3 facilitators are indicated with an asterisk. Additional text has been added to some items based on the focus group recording that gives context to the item being raised and can be seen within square brackets.

| Theme | Subtheme | Items |
|---|---|---|
| Distrust | Anonymity / Confidentiality | Anonymity (of participation) |
| | | Lack of trust of confidentiality of data |
| | | Don't want to share medical/personal information |
| | Impact of Research | Not knowing if the research will benefit me or others - [2nd] |
| | | Not being sensitized to the importance of such research - [2nd] |
| | | Implications for not taking pharmaceutical drugs* |
| | | Lack of trust that it will lead to anything* |
| | | Fear of toxicity/ side effects of treatments |
| | | Poor understanding of how I can influence change/help the research |
| | | Fear of addiction to pain treatments |
| | | Don't think research will make any difference |
| | | A lack of seeing where the research can lead / not getting feedback |
| | Professionals / Setting | Lack of understanding about chronic pain - [1st] |
| | | Fear of scrutiny - [2nd] |
| | | Fear about what might be involved - [3rd] |
| | | Fear surrounding group situations - [3rd] |
| | | Previous negative experiences of taking part - [3rd] |
| | | Fear of the research process* |
| | | Lack of trust of health professionals/researchers* |
| | | Doctors not being interested in new or ongoing research* |
| | | Distrust of medical professionals* |
| | | Fear of judgment (friends, family, professionals)* |
| | | Not wanting to talk about pain* |
| | | General barriers to alternative treatments |
| | | Concern of invalidation |
| | | Fear of being judged |
| | | Lack of belief about chronic pain |
| | | Fear of judgement/stigma - people will think I am making the pain up, or not being able to relate |
| | | Fear of sharing |
| | | Judgement (for having chronic pain) |
| | | Not wanting to talk about upsetting things |
| | | Feelings of embarrassment |
| | | Stigma around pain and disability |

| | | |
|---|---|---|
| | | Fear of invalidation of pain |
| | | Invisibility of chronic pain |
| Lack of Accessibility / Physical Practicalities | Travel | Travel |
| | | Location of research studies (other locations/hard to travel to) |
| | | Travel and pain that could get worse with travel |
| | | Is it possible to travel to location? |
| | | Winter - bad weather will stop people going out |
| | | Travel issues |
| | Accessibility - Personal | Environments not conducive to research, particularly in public places/unknown environment - 3rd |
| | | Accessibility of the research placement - 3rd |
| | | Fear of losing control [of pain management] when participating (avoidance behaviours) |
| | | Is venue accessible once there? |
| | | Issues for attending in person |
| | | Accessibility of research psychologically |
| | | Lack of childcare |
| | Accessibility – Technological | Not having internet - 2nd |
| | | Not having necessary equipment (e.g. laptop) - 3rd |
| | | Lack of access to technology - 3rd |
| | | Communication - visual and audio options (accessibility for those with additional needs and anyone with chronic pain) |
| | | Access issues for technology |
| | | Fearful of online tools |
| | | Not having access to technology to participate |
| | Time | Not having time / cannot make dates offered - 3rd |
| | | Limited time to take part - 3rd |
| | | Being asked to participate at inappropriate times* |
| | | Not having time to take part |
| | | Lack of time |
| Chronic Symptoms & Comorbidities | Fatigue | Worry of future fatigue (as a consequence of taking part) - 1st |
| | | Not having energy - 2nd |
| | | Fatigue during research - 3rd |
| | | Too much fatigue to take part - 3rd |
| | | Not being able to commit to the research on the day [not enough energy]* |
| | | Too much fatigue |
| | Psychological Symptoms | Mental health issues - 3rd |
| | | Psychological/social difficulties associated with pain (difficulty talking about psychological consequences) - 3rd |
| | | In too much pain (physically/mentally)* |
| | | Lowered mood reduces motivation to take part |
| | Physical Symptoms | Unpredictability of chronic pain (impact of pain on being able to participate due to fluctuation) - 1st |
| | | Fear of increase in pain (in other areas) - 2nd |
| | | Being too unwell - 3rd |

| | | |
|---|---|---|
| | | Being in too much pain to consider being involved in research - 3rd |
| | | In too much pain (physically/mentally)* |
| | | Being in too much pain to take part |
| | | Pain preventing people taking part - how will I cope with participation? |
| | | Worry pain will get worse |
| | | Using hands to take part |
| | | Taking part could exacerbate the pain |
| Lack of Information | Study Details | Not knowing research expectations (of you) - 3rd |
| | | Not knowing what to expect |
| | | Lack of information to know what to expect |
| | | Lack of understanding about non-pharmaceutical treatments |
| | Recruitment / Study Advertisement | Little or no awareness of research happening/Lack of advertising of research - 1st |
| | | Not knowing about the research because it is not often spoken about - 1st |
| | | Not being aware that research takes place/ not being mentioned in doctors' surgery - 1st |
| | | Not knowing what research is out there - 2nd |
| | | Unaware that there is anything beyond pharmaceutical - 2nd |
| | | Researchers not reaching out into communities - 2nd |
| | | Not knowing the research exists - 2nd |
| | | Not knowing that there are non-pharmacological alternatives - 2nd |
| | | Lack of information about how to participate / fear of unknown |
| | | Lack of contact information to take part |
| | | Don't want to take medication so avoid pain clinics etc [and therefore won't see advertisements] |
| | | Not knowing where to find out about research studies |
| Lack of Motivation | Incentivisation / Compensation | Lack of incentives to take part - 1st |
| | | Doesn't help pain right now - 2nd |
| | | Needing expenses for travel etc* |
| | | Lack of financial renumeration |
| | | Travel renumeration not coming before |
| | | Compensation (must be worth it) |
| | Lack of Priority | Not a priority because of other pain - 1st |
| | | Caring responsibilities being the focus - 2nd |
| | | Burden: Not motivated to take extra responsibility - participation too much to do on top of pain management - 3rd |
| | | Not a priority for managing time - 3rd |
| | | Lack of motivation - 3rd |
| | | Only focus on pain during crisis rather than prevention and management |
| | | Lack of interest |
| Cultural Barriers / Individual Differences | | Lack of cultural understanding about experience of pain* |

| | |
|---|---|
| | Cultural differences in pain management/labelling* |
| | Not taking into account individual differences* |
| | Lack of culturally specific approaches in research |
| | Learning disabilities and sensory issues |
| | Lack of cultural specific approaches in research |
| Self-Identification / Eligibility | Not feeling like you can take part if you don't have a diagnosis - 2nd |
| | Lack of diagnosis - 3rd |
| | Feel that pain is not bad enough, so feel that they should not attend* |
| | Not being "poorly" enough to take part |
| | Denial |
| | Thinking pain is just a normal part of ageing |

**S3 Table of Facilitators from Phase 1**

*Table S3.* Themes and subthemes for facilitator items, with the topmost facilitators from a focus group indicated with a "1st", second topmost facilitators indicated with a "2nd", and third topmost facilitators indicated with a "3rd". Items which received votes but did not rank within the top 3 facilitators are indicated with an asterisk.

| **Theme** | **subtheme** | Item |
|---|---|---|
| Improved Accessibility | Practical Accessibility | Extra support - 1st |
| | | Accessibility to research - 3rd |
| | | Easy access to venue/internet - make it a local venue that is easy to reach and access* |
| | | Having accessible research* |
| | | Accessibility of location |
| | | Public transport available |
| | | Childcare options (in person testing) |
| | | Access to pain relief/management at an in-person session |
| | | Making in person research physically accessibly |
| | | Offering assistance with technology |
| | Timings | Flexibility in when to participate (around pain) - 2nd |
| | | Sweet spot' of pain level at the time - 3rd |
| | | Flexibility for time of involvement - 3rd |
| | | Shorter focus groups (around one hour) / breaks if longer sessions* |
| | | Having serval options for times of sessions / out of hours testing* |
| | | Out of hours participation* |
| | | Give data when the participant is able* |
| | | Several options of dates/times for sessions |
| | | Can be done in my own time |
| | | Flexibility of times for research |
| | | One hour as maximum (depends upon individual needs) |
| | Participation Options | Having both online and in person option available - 1st |
| | | Remote participation (online) - 2nd |
| | | More ways to participate (especially online; Zoom/Google Forms/Survey Monkey) - 2nd |
| | | Flexibility of methods of research (shorter and longer options) - 3rd |
| | | Preference for zoom - 3rd |
| | | Flexible options about how to participate (paper/computer/phone)* |
| | | Option to have researchers come to you* |
| | | Options of where to go for the research* |
| | | Flexibility for unpredictability (range of times as well as formats for participation) |
| | | Different formats available to participate (e.g. paper vs. talking) |
| | | Having a range of contribution methods (paper, telephone) |

| | | |
|---|---|---|
| | Communication / Advertisement | Putting information in community and medical spaces - 1st |
| | | Awareness of the opportunities/More advertising through trusted routes, e.g. NHS/GP surgery/ - 1st |
| | | Hear about research from GP/ trusted person - 3rd |
| | | Hearing about research in trusted setting* |
| | | Calls for taking part being easy to find and available to a wide range of people* |
| | | Research information needs to be accessible (understood) |
| | | Better communication (visual & audio - accessibility) |
| | | Availability in different languages |
| | | Clear publicising of research |
| | | Better advertising: Sharing social media posts / Word of Mouth (Facebook / Twitter)<br>Advertising the research |
| | | Advertising on social media |
| Positive Impact of Participation | Impact on Day | Improvement in pain - 2nd |
| | | Knowing there are others who experience the issues - 2nd |
| | | Feeling a part of the community (others experiencing same things) / loneliness - 3rd |
| | | Understand how it might impact pain on the day |
| | | Knowing it won't influence my current pain levels |
| | | Participating in a group |
| | Impact After | Research could facilitate potential new methods - 2nd |
| | | Knowing if research can have long term benefits - 3rd |
| | | More information about the benefits of the research - 3rd |
| | | Get feedback about the research outputs (results)/ accessible - 3rd |
| | | Better understanding of how their contribution can help* |
| | | If the research can improve personal wellbeing* |
| | | Have an idea about the possible outcomes of the research* |
| | | Getting feedback about outcomes and a thank you for taking part* |
| | | Follow up about what happens with the research*<br>Knowing if the research will have medical implications* |
| Detailed & Accessible Information | Eligibility | Being clear about diagnoses that are relevant - 3rd |
| | | Being able to invite other participants to take part/ knowing people who have taken part |
| | Research | Very detailed information about what to expect - 1st |
| | | Knowing research aims - 2nd<br>Clarity about group or one on one sessions - 3rd |
| | | More information about the research and benefits - 3rd |
| | | Clarity of what to expect* |
| | | Confidence that the research is well founded* |
| | | More information about what is involved* |
| | | Being clear as to what adjustments can be made for the research |
| | | Clarity how to participate (online/offline) |
| | | Having more information about what the research is based on |

| | |
|---|---|
| | More information needed regarding what the research involves, aims, rationale |
| | Understanding existing research |
| | Knowing what will happen on the day (reduce anxiety) |
| Increased Motivation | Financial compensation for your time for participation - [1st] |
| | Incentive for taking part (financial/ other) - [1st] |
| | Improvement in pain - [2nd] |
| | Research needs to be interesting and relevant - [2nd] |
| | Paying people properly for their time in research - [3rd] |
| | Easier access to healthcare - [3rd] |
| | Paying people properly for their time in research - [3rd] |
| | Reimbursement of travel expenses* |
| | Having incentives for taking part |
| | Altruistic motivations (helping others) |
| | Wider acceptance of non-pharmacological interventions |
| Safe Space | Approachable researchers - [3rd] |
| | Smaller focus groups more comfortable / discuss in safe space / anonymity - [3rd] |
| | Non-clinical/ non-academic setting for the study* |
| | Having researchers with lived experience* |
| | Discussion between researcher and participant (individualised) prior to participation to cater for needs* |
| | Feeling like others (researchers/professionals) care |
| | Understanding there is help available |
| | Videos of people involved/ researchers |
| | Being able to have a companion during the research |
| | Having researchers who are understanding |

**S4 Table of chronic pain conditions prevalent in phase 2**

*Table S4.* Prevalence of chronic pain condition within survey participant sample. Note: Several participants had more than one chronic pain condition, therefore the total of the percentages does not equal the total number of participants. *Unspecified is used for participants who either did not specify a specific condition or said they were awaiting diagnosis.

| Chronic Pain Condition | Prevalence within Participant Sample |
|---|---|
| Rheumatoid Arthritis | 37% |
| Fibromyalgia | 25% |
| Chronic Back pain | 19% |
| Osteoarthritis | 12% |
| Nerve Pain | 10% |
| Arthritis | 7% |
| Ehlers Danlos Syndrome | 5% |
| Chronic Hip Pain | 5% |
| Psoriatic Arthritis | 4% |
| Endometriosis | 3% |
| M.E. | 3% |
| Unspecified* | 3% |
| Hypermobility | 3% |
| Chronic Leg Pain | 2% |
| Migraine | 2% |
| Scoliosis | 2% |
| Chronic Knee Pain | 2% |
| Sjogren’s Syndrome | 2% |
| Cervical Spondylosis | 2% |
| PCOS | 2% |
| Chronic Neck Pain | 2% |
| Chronic Fatigue Syndrome | 2% |
| Chronic Shoulder Pain | 2% |
| Chronic Pain Syndrome | 2% |
| Hyperthyroidism | 1% |
| Polyarthralgia | 1% |
| Juvenile Idiopathic Arthritis | 1% |
| Scleritis | 1% |
| Idiopathic Musculoskeletal | 1% |
| Polymyalgia | 1% |
| Chronic Bladder Pain | 1% |
| Temporomandibular Joint Disorder | 1% |
| Osteoporosis | 1% |
| Tendonitis | 1% |
| Irritable Bowel Syndrome | 1% |
| Gastroparesis | 1% |
| Pancreatitis | 1% |
| Bursitis | 1% |
| Vulvodynia | 1% |
| Chiari Malformation | 1% |
| Syringomyelia | 1% |
| Muscular Dysfunction | 1% |
| Idiopathic Intracranial Hypertension | 1% |
| Trigeminal Neuralgia | 1% |

**S5 Exploratory Phase 2 Analyses**

Within the survey, participants were asked to rank the level of their agreement / disagreement on a Likert scale from -3 to +3, allowing for a more in depth look at the nature of the opinions that participants held regarding the barrier and facilitator themes gained within phase 1. Exploratory data presentation can be seen in Figure S3.

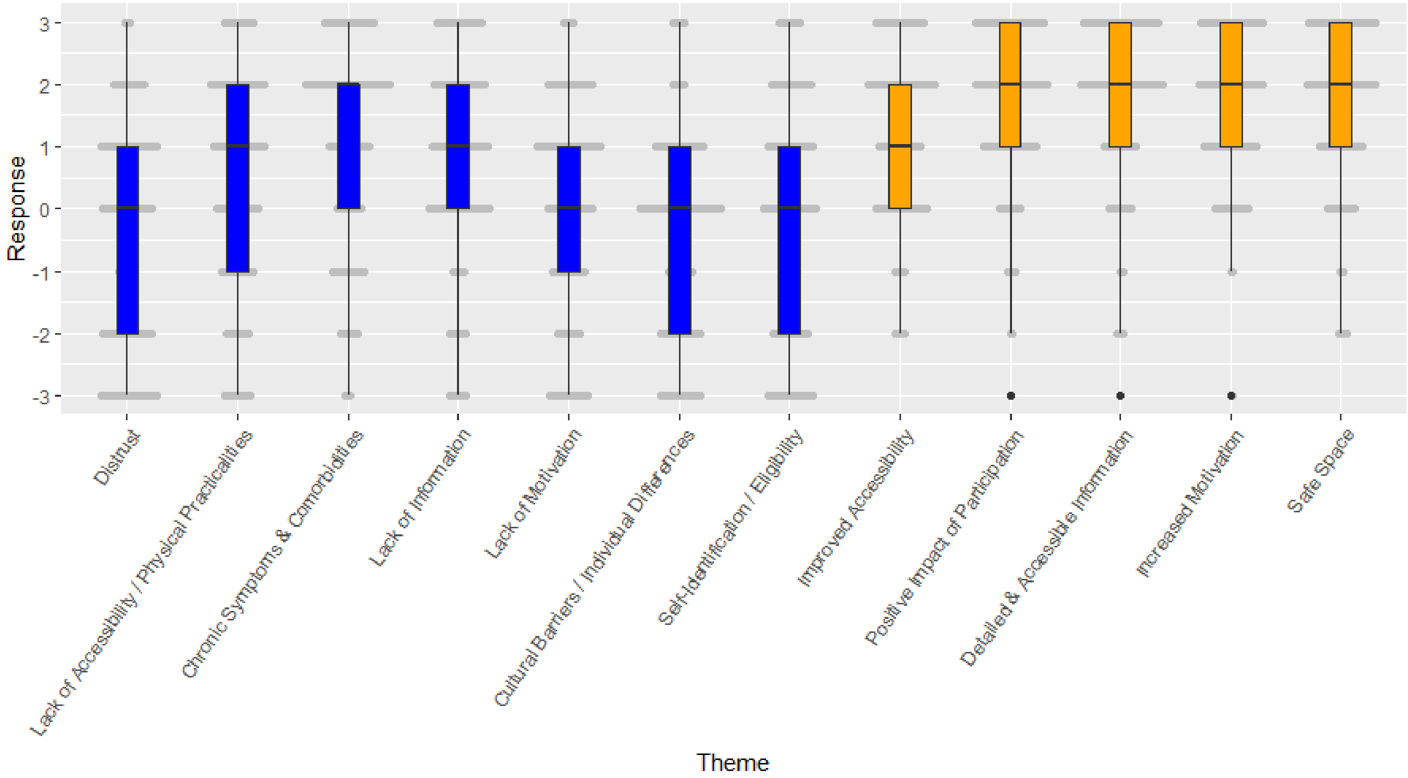


*S3.* Boxplot with jitter showing distribution of Likert scale responses regarding the strength of agreement, disagreement, or neutral opinion of themes. +3 indicates strong agreement, 0 indicates a neutral opinion and -3 indicates strong disagreement with the theme. Blue bars represent Barriers, Orange bars represent Facilitators. Jitter can be seen in grey, with outliers shown in black.
