# Supplementary material for: Understanding barriers and facilitators to non-pharmaceutical chronic pain research engagement among people living with chronic pain in the UK: a two-phase mixed-methods approach
Source: BMJ Open. 2024 Dec 22;14(12):e089676. doi: 10.1136/bmjopen-2024-089676 (PMC11751962; doi:10.1136/bmjopen-2024-089676)
Supplement: Supplementary file 3 [file bmjopen-2024-089676.reviewer_comments.pdf]

PEER REVIEW HISTORY

BMJ Open publishes all reviews undertaken for accepted manuscripts. Reviewers are asked to complete a checklist review form and are provided with free text boxes to elaborate on their assessment. These free text comments are reproduced below.

ARTICLE DETAILS

Title (Provisional)

Understanding Barriers and Facilitators to Non-Pharmaceutical Chronic Pain Research Engagement Amongst People Living with Chronic Pain in the UK: A two-phase mixed methods approach.

Authors

Hansford, Kirralise; Crossland, Anna E.; McKenzie, Kirsten J; Baker, Daniel; Preston, Catherine

---

VERSION 1 - REVIEW

**Reviewer**	**1**
**Name**	**Lopatina, Elena**
**Affiliation**	**University of Calgary**
**Date**	**16-Jul-2024**
**COI**	**No competing interests**

Thank you for the opportunity to review this interesting work. This is an important topic, especially in light of the prevalence and burden of chronic pain. The authors used appropriate methods, and the manuscript is well-written, making it a pleasure to review.

I have a few suggestions for the authors:

1. The authors state that this is a mixed-methods research study, however, the manuscript does not explicitly discuss the triangulation approaches used, which are essential in mixed-methods research. Adding details on the triangulation will further strengthen this work.

2. In the introduction, the authors mention that phase 2 was conducted “to assess the generalizability of the facilitator and barrier themes to the wider UK population with chronic pain.” It would be helpful to discuss whether the phase 2 sample was indeed representative of the wider UK population and how this might have affected the findings. Given the homogeneity of the samples, an in-depth discussion on how the identified themes would apply to a more diverse population would be interesting.

3. I am curious to what extent the description of the barriers and facilitators themes in phase 2 could affect the agreement and disagreement results. For example, the description of

"Distrust" as “ having distrust of the level of anonymity and confidentiality, or having a distrust of medical or research professionals” and included examples “Distrust of anonymity /confidentiality; Distrust of the impact of the research; Distrust of medical / research professionals and settings” do not seem to capture the full complexity of the theme. It would be beneficial to provide more details on how the research team developed these definitions and whether they were piloted in any way. Additionally, a discussion about the potential limitations of how the themes were presented and their implications would be helpful.

**Reviewer**	**2**
**Name**	**McNeilage, Amy**
**Affiliation**	**The University of Sydney**
**Date**	**24-Sep-2024**
**COI**	**None to declare**

Overview:

- This is a well-designed mixed methods study examining barriers to participating in non-pharmacological pain treatment research. Given some of the recent discussion around the challenges of recruiting patients with CNCP to clinical trials, this is a valuable and timely contribution to the research literature.

Introduction:

- The introduction seems to be missing some important literature supporting the rationale for the study. For instance, several research groups have published papers outlining the challenges they’ve faced recruiting patients to their clinical trials. The introduction would be strengthened by evidence that it is indeed difficult to recruit patients to these studies in addition to some of the (already provided) reasons why this might be the case. The authors have also outlined the reasons why it is important to involve patients in designing novel treatments. However, they have not discussed the potential risks/drawbacks of participation barriers (e.g., certain types of participants might be overrepresented; failure to reach required sample size to evaluate efficacy, etc.). The authors should consider strengthening the rationale provided for the study.

- It is not clear how/why the authors reached the stated hypotheses? Transportation and efficacy concerns are mentioned earlier in the introduction, but so are other factors (not included in the hypotheses) such as lack of knowledge about research, concerns about side effects, and distrust. Why were some included in the hypotheses and not others? Further, why were there no hypotheses for facilitators given it is stated that prior research has found

facilitators to include things such as advancement of science, seeking pain relief, and receiving compensation?

Methods:

- Was the informed consent provided verbally or in writing?

- The data analysis approach described in the methods does not seem consistent with a thematic analysis. As I understand, it involved sorting very thin data (i.e., several word “items”) into categories and then ranking the categories according to participant ratings. This method is more akin to a content analysis or other coding frameworks that focus on frequency and categorisation of text, rather than identifying deeper, more nuanced themes. It does seem that the data is rich enough to support a thematic analysis. Moreover, providing statistics (i.e., Cohen’s Kappa) for agreement on themes is inconsistent with most qualitative approaches and not something Braun and Clarke suggest for thematic analysis. I recommend that the authors reconsider their use of the term 'thematic analysis' and provide a more accurate description of their methodology to better align with the approach they have used.

Discussion:

- This sentence requires a citation: “… which is likely a further barrier for those from ethnically diverse backgrounds where diagnoses may not be as prevalent”

- This statement requires some elaboration: “Considering the impact that research participation can have on individuals with chronic pain - from physical impact, time commitments, and psychological impacts - compensation and additional incentives must be given to participants”. Providing financial incentives to research participants is not a straightforward matter. While compensation is important to acknowledge participants' time, effort, and potential discomfort, it must be carefully considered to avoid ethical concerns such as coercion or undue influence. Moreover, the recent emergence of 'imposter participants' - individuals who misrepresent themselves to participate in studies for financial gain - raises additional concerns about data validity and participant screening. Striking the right balance between fair compensation and maintaining the integrity of the research process is crucial, especially in sensitive populations like individuals with chronic pain.

- There is no discussion of study limitations, which include the constraints of convenience sampling as well as the lack of patient involvement in the design, conduct, and reporting, among other things.

**Reviewer**	**3**
**Name**	**Cetin, Hacer**
**Affiliation**	**Mersin Universitesi, Pediatric Nursing**
**Date**	**01-Oct-2024**

COI **No there isnot**

The author has worked in detail with this research and reached results that will constitute evidence. This publication has been evaluated as a very successful work.

****** The reviewer provided a marked copy with additional comments. Please contact the publisher for full details.**

VERSION 1 - AUTHOR RESPONSE

Reviewer: 1
Dr. Elena Lopatina, University of Calgary
Comments to the Author:
Thank you for the opportunity to review this interesting work. This is an important topic, especially in light of the prevalence and burden of chronic pain. The authors used appropriate methods, and the manuscript is well-written, making it a pleasure to review.

I have a few suggestions for the authors:
1. The authors state that this is a mixed-methods research study, however, the manuscript does not explicitly discuss the triangulation approaches used, which are essential in mixed-methods research. Adding details on the triangulation will further strengthen this work.

Details regarding the triangulation approaches used have been added to a new section below section 2 Manuscript structure and are as follows:

"Triangulation between the qualitative and quantitative data within this report was approached sequentially at the design stage, whereby the findings from the qualitative data in Phase 1 were used to create materials for the quantitative data in Phase 2. Qualitative and quantitative data were then merged during the manuscript's discussion and integrated via written narrative to allow for a holistic approach to understanding which barriers and facilitators exist for people when considering taking part in non-pharmaceutical chronic pain research."

2. In the introduction, the authors mention that phase 2 was conducted "to assess the generalizability of the facilitator and barrier themes to the wider UK population with chronic pain." It would be helpful to discuss whether the phase 2 sample was indeed representative of the wider UK population and how this might have affected the findings. Given the homogeneity of the samples, an in-depth discussion on how the identified themes would apply to a more diverse population would be interesting.

Additional text has been added to the discussion about the limited diversity of the phase 2 sample despite this being one of the aims of phase 2, which can be seen in the text below. We have chosen not to include an in-depth discussion of how the identified themes would apply to a more diverse population, since we did not gather detailed enough data within the focus groups to make these comments, and we do not wish to speculate as to what minority ethnic groups in the UK would

experience as barriers. Instead we make it clear that more research in this area is needed to be able to have these discussions.

"There were some methodological limitations present in the current study, such as the use of online measures to collect both focus group and questionnaire data, which despite being used to try and encourage participation from as many people as possible, would have prevented people without internet access from taking part. Additionally, despite Phase 2 having a much larger sample size than Phase 1, the aim of this phase was to gather data from a wider and more diverse sample than that collected in Phase 1. Unfortunately, the data collected in both phases were almost exclusively from people of a white ethnic background, which means that generalising these findings to people from other ethnic backgrounds might be difficult and highlights the need for more research into barriers and facilitators to participation from minoritised or marginalised ethnic groups within the UK. It is possible that the lack of inclusion of a diverse range of interested parties in the design of the study could have contributed to this overrepresentation of white women within the sample, therefore it is highly recommended that future research includes contributions from, and collaboration with, people from a wider range of communities and ethnic backgrounds in the design and advertisement of potential projects. One final methodological concern relates to the statements used in the Phase 2 questionnaire. These statements were derived from the items generated within Phase 1, but since so many items were generated, it was not possible to list all of them for the purposes of the questionnaire. Therefore, it is possible that the levels of agreement and disagreement with the themes could have differed if all items were presented to Phase 2 participants. Due to time constraints, this was not the approach used, but it is important to note that Phase 2 data relates directly to the statement definitions of the themes (which can be seen in Appendix A), rather than the complete lists provided in Tables 2 and 3."

3. I am curious to what extent the description of the barriers and facilitators themes in phase 2 could affect the agreement and disagreement results. For example, the description of "Distrust" as " having distrust of the level of anonymity and confidentiality, or having a distrust of medical or research professionals" and included examples "Distrust of anonymity /confidentiality; Distrust of the impact of the research; Distrust of medical / research professionals and settings" do not seem to capture the full complexity of the theme. It would be beneficial to provide more details on how the research team developed these definitions and whether they were piloted in any way. Additionally, a discussion about the potential limitations of how the themes were presented and their implications would be helpful.

This is a very worthwhile point raised, and we have made sure to include some text about this in the phase 2 data collection and the limitations section of the discussion which can be seen below:

Data collection section - "Researchers KH and CP collectively decided on the statements to give for each theme, making sure to include as much detail from the items as possible without making the statements too long for participants to read."

Discussion - "One final methodological concern relates to the statements used in the Phase 2 questionnaire. These statements were derived from the items generated within Phase 1, but since so many items were generated, it was not possible to list all of them for the purposes of the

questionnaire. Therefore, it is possible that the levels of agreement and disagreement with the themes could have differed if all items were presented to Phase 2 participants. Due to time constraints, this was not the approach used, but it is important to note that Phase 2 data relates directly to the statement definitions of the themes (which can be seen in Appendix A), rather than the complete lists provided in Tables 2 and 3."

Reviewer: 2
Ms. Amy McNeilage, The University of Sydney
Comments to the Author:
Overview:
- This is a well-designed mixed methods study examining barriers to participating in non-pharmacological pain treatment research. Given some of the recent discussion around the challenges of recruiting patients with CNCP to clinical trials, this is a valuable and timely contribution to the research literature.

Introduction:
- The introduction seems to be missing some important literature supporting the rationale for the study. For instance, several research groups have published papers outlining the challenges they've faced recruiting patients to their clinical trials. The introduction would be strengthened by evidence that it is indeed difficult to recruit patients to these studies in addition to some of the (already provided) reasons why this might be the case. The authors have also outlined the reasons why it is important to involve patients in designing novel treatments. However, they have not discussed the potential risks/drawbacks of participation barriers (e.g., certain types of participants might be overrepresented; failure to reach required sample size to evaluate efficacy, etc.). The authors should consider strengthening the rationale provided for the study.

Additional literature has been added to the introduction around difficulty recruiting participants and some of the issues with participant heterogeneity within UK health samples, which can be seen below:

"In 2018, national standards were set for patient engagement in research, highlighting the importance of including patients when designing novel treatments (NICE, 2018). Patient engagement can lead to improved credibility of results, and improvements in direct applications to patient samples (Domecq et al., 2014), therefore, understanding barriers and facilitators for patient involvement is paramount. However, achieving meaningful patient involvement in research and clinical trials can be a difficult process, with reports of around 37% of projects having a reduced sample size than planned and 11% of clinical research failing to recruit any participants at all (Chaudhari et al., 2020). Additionally, the people who do participate in research are often from homogeneous demographics, with a 2021 report of people who take part in health research in the UK finding that participants are predominantly female, white, heterosexual, and aged over 61 years (NIHR, 2021)."
- It is not clear how/why the authors reached the stated hypotheses? Transportation and efficacy concerns are mentioned earlier in the introduction, but so are other factors (not included in the

hypotheses) such as lack of knowledge about research, concerns about side effects, and distrust. Why were some included in the hypotheses and not others? Further, why were there no hypotheses for facilitators given it is stated that prior research has found facilitators to include things such as advancement of science, seeking pain relief, and receiving compensation?

Clarity has been added for the reason why each hypothesis was made or not made, which can be seen below:

"For phase 1, our preregistered hypotheses were based on the most prevalent barriers mentioned in existing literature, which were that (i) transportation problems and (ii) the concerns regarding the efficacy of non-pharmaceutical treatments, would arise as top-most barriers. Since previous research was more limited for facilitators to taking part in research, there were no directional hypotheses regarding facilitators. We had no directional hypotheses for phase 2."

Methods:
- Was the informed consent provided verbally or in writing?

This has been included:

"All participants gave informed written consent using an online form to take part in their focus group. At the start of each focus group participants were reminded that they could withdraw at any time, and they were told when the recording of the focus group would take place."

- The data analysis approach described in the methods does not seem consistent with a thematic analysis. As I understand, it involved sorting very thin data (i.e., several word "items") into categories and then ranking the categories according to participant ratings. This method is more akin to a content analysis or other coding frameworks that focus on frequency and categorisation of text, rather than identifying deeper, more nuanced themes. It does seem that the data is rich enough to support a thematic analysis. Moreover, providing statistics (i.e., Cohen's Kappa) for agreement on themes is inconsistent with most qualitative approaches and not something Braun and Clarke suggest for thematic analysis. I recommend that the authors reconsider their use of the term 'thematic analysis' and provide a more accurate description of their methodology to better align with the approach they have used.

The thematic analysis did consist of sorting the item data into codes and themes as mentioned, but these were not ranked according to the ratings given by participants. As mentioned in the manuscript the ratings were used to rank items within a theme, but the order of the themes was generated by simply giving the largest (containing the most items) first. The thematic analysis used is in line with the nominal group technique approach used to collect data, which has been used in previous focus group research to generate data for thematic analysis (McMillan et al., 2014 – this reference has also been added to the manuscript to support the use of thematic analysis within the NGT for large datasets (section 3.5 Data analysis)). The statistics provided for the agreement with themes between researchers was given to show consistency between researchers. Additional text has been added to the data analysis section to aid with understanding of the analysis process, as can be seen below:

"To facilitate comparisons of items from each focus group across all sessions, researchers used thematic analysis through a deductive approach (Braun & Clark, 2006) to categorise items into either facilitator or barrier themes based on a consensus of interpretation of the item meanings. A thematic

analysis approach can be employed when generating large data with the NGT (McMillan et al., 2014), therefore this was the approach used here. A post-positivist approach was used for thematic analysis, to focus on the individual experiences of people living with chronic pain when considering taking part in non-pharmaceutical research (Kiger & Varpio, 2020). Through these approaches, three researchers (KH, AC, and CP) independently reviewed the raw data for familiarisation, before identifying patterns within the data set. They then created codes for each group of items, before placing these coded items into overarching themes within either the barrier or facilitator structure. An additional fourth researcher (KM) was included to facilitate discussion of theme review amongst the three researchers. Following this, 2 researchers (KH & CP) refined and defined the themes with the following agreements; Barrier themes percentage agreement: 87.03% (Berk, 1979; McDermott, 1988), Barrier themes Cohen's Kappa: 0.832 (strong agreement; McHugh, 2012; near perfect agreement; Landis, 1977), Facilitator themes percentage agreement: 89% (Berk, 1979; McDermott, 1988), Facilitator themes Cohen's Kappa: 0.869 (strong agreement; McHugh, 2012; near perfect agreement; Landis, 1977). This process resulted in the final list of themes agreed upon by all 4 researchers. Themes were sorted by size, with the theme containing the most items presented first. Participant ratings of items were tallied to give topmost items within each theme (McMillan et al., 2014)."

As per another reviewers comment, an additional section has also been added regarding the triangulation approached used to integrate the qualitative and quantitative data, further aiding understanding of the data analysis and methodological approaches used within this manuscript:

"Triangulation between the qualitative and quantitative data within this report was approached sequentially at the design stage, whereby the findings from Phase 1's qualitative data were used to create materials for Phase 2's quantitative questionnaire. Qualitative and quantitative data are then merged during the manuscript's discussion and integrated via written narrative to allow for a holistic approach to understanding what barriers and facilitators exist for people when considering taking part in non-pharmaceutical chronic pain research."

Discussion:
- This sentence requires a citation: "… which is likely a further barrier for those from ethnically diverse backgrounds where diagnoses may not be as prevalent"

A reference has been added and the text has been slightly changed:

"…which is likely a further barrier for those from ethnically diverse backgrounds where diagnoses may show different prevalence rates (Versus Arthritis, 2017)."

- This statement requires some elaboration: "Considering the impact that research participation can have on individuals with chronic pain - from physical impact, time commitments, and psychological impacts - compensation and additional incentives must be given to participants". Providing financial incentives to research participants is not a straightforward matter. While compensation is important to acknowledge participants' time, effort, and potential discomfort, it must be carefully considered to avoid ethical concerns such as coercion or undue influence. Moreover, the recent emergence of 'imposter participants' - individuals who misrepresent themselves to participate in studies for financial gain - raises additional concerns about data validity and participant screening. Striking the right balance between fair compensation and

maintaining the integrity of the research process is crucial, especially in sensitive populations like individuals with chronic pain.

This point has been added to the text around participant payments, with notes on imposter participants and the use of large language models during focus group projects as can be seen below:

"Considering the impact that research participation can have on individuals with chronic pain - from physical impact, time commitments, and psychological impacts - compensation and additional incentives must be given to participants. The facilitator theme "Increased Motivation" demonstrates that compensation can come from sources such as financial renumeration, pain improvement, travel reimbursement, and altruistic motivations. The facilitator theme "Positive Impact of Participation" highlights that feeling part of a community can also have long term benefits. However there have been issues with compensation for research becoming an incentive for "imposter participants"; those who are motivated to take part in research for financial gain and represent themselves inauthentically. For example, a recent study commented on large language models being used by imposter participants to give detailed qualitative responses during focus group research projects (Stafford, 2024). Therefore, it is important to consider trusted routes for research advertisement to try and avoid such imposters diluting clinical data. "

- There is no discussion of study limitations, which include the constraints of convenience sampling as well as the lack of patient involvement in the design, conduct, and reporting, among other things.

A paragraph has been added to the discussion specifically relating to limitations of the study, including notes about the lack of involvement of stakeholders, as can be seen below:

"There were some methodological limitations present in the current study, such as the use of online measures to collect both focus group and questionnaire data, which despite being used to try and encourage participation from as many people as possible, would have prevented people without internet access from taking part. Additionally, despite Phase 2 having a much larger sample size than Phase 1, the aim of this phase was to gather data from a wider and more diverse sample than that collected in Phase 1. Unfortunately, the data collected in both phases were almost exclusively from people of a white ethnic background, which means that generalising these findings to people from other ethnic backgrounds might be difficult and highlights the need for more research into barriers and facilitators to participation from minoritised or marginalised ethnic groups within the UK. It is possible that the lack of inclusion of a diverse range of interested parties in the design of the study could have contributed to this overrepresentation of white women within the sample, therefore it is highly recommended that future research includes contributions from, and collaboration with, people from a wider range of communities and ethnic backgrounds in the design and advertisement of potential projects. One final methodological concern relates to the statements used in the Phase 2 questionnaire. These statements were derived from the items generated within Phase 1, but since so many items were generated, it was not possible to list all of them for the purposes of the questionnaire. Therefore, it is possible that the levels of agreement and disagreement with the themes could have differed if all items were presented to Phase 2 participants. Due to time constraints, this was not the approach used, but it is important to note that Phase 2 data relates directly to the statement definitions of the themes (which can be seen in Appendix A), rather than the complete lists provided in Tables 2 and 3."

Reviewer: 3
Dr. Hacer Cetin, Mersin Universitesi, Mersin Universitesi
Comments to the Author:
The author has worked in detail with this research and reached results that will constitute evidence. This publication has been evaluated as a very successful work.

VERSION 2 - REVIEW

**Reviewer**	**2**
**Name**	**McNeilage, Amy**
**Affiliation**	**The University of Sydney**
**Date**	**18-Nov-2024**
**COI**	

I have reviewed the authors' responses to my prior comments, and I am largely satisfied with their changes. I think they have improved the manuscript in a number of areas. I am still slightly uncomfortable with this approach being labelled a thematic analysis (particularly given they cite Braun and Clarke's approach) but I'll leave that to the editor's discretion. I have one minor comment on the revisions. I am confused by the new reference to the "Delphi interview technique" in section 5.2 as this is not referred to anywhere else in the manuscript. It does not appear to me that Phase 2 used a Delphi interview technique. If it did, I think this requires further detailing in the methods. If the editor is satisfied with the authors' explanation of this, I am comfortable recommending acceptance. Finally, I think the manuscript would benefit from some copyediting. There are many instances where two or more sentences have been combined in a single sentence (separated by a comma). This would improve the readability of the manuscript.

VERSION 2 - AUTHOR RESPONSE

Reviewer's comments:

I have one minor comment on the revisions. I am confused by the new reference to the "Delphi interview technique" in section 5.2 as this is not referred to anywhere else in the manuscript. It does not appear to me that Phase 2 used a Delphi interview technique. If it did, I think this requires further detailing in the methods. If the editor is satisfied with the authors' explanation of this, I am comfortable recommending acceptance.

Further detail has now been given in Phase 2's methods of how the technique used within this study relates to the Delphi technique, which was not used as iterations of qualitative data collection, but rather through using the qualitative data generated in phase 1 to create the questionnaire which generated quantitative data for phase 2. A reference has been added to this section to give support for this technique and previous mention of the Delphi technique in section 5.2 (now correctly numbered 6.2) links readers to section 6.4 where this new text has been added:

"After running the focus groups, the Delphi technique was used through creating a questionnaire containing all themes regarding both barriers and facilitators to taking part in non-pharmacological chronic pain research. This study employed one of the most common approaches to the Delphi technique [20] through using data from Phase 1's qualitative approach to form the questionnaire items for the subsequent quantitative data generation within Phase 2."

Finally, I think the manuscript would benefit from some copyediting. There are many instances where two or more sentences have been combined in a single sentence (separated by a comma). This would improve the readability of the manuscript.

The manuscript has been read over and some comma's have been removed to try and aid readability.
